# The relationship between interoception and agency and its modulation by heartbeats: an exploratory study

**DOI:** 10.1038/s41598-022-16569-6

**Published:** 2022-08-10

**Authors:** Akihiro Koreki, Diana Goeta, Lucia Ricciardi, Talia Eilon, Jiaying Chen, Hugo D. Critchley, Sarah N. Garfinkel, Mark Edwards, Mahinda Yogarajah

**Affiliations:** 1 Neurosciences Research Centre, St George’s University of London, London, UK; 2 Department of Psychiatry, National Hospital Organization Shimofusa Psychiatric Medical Center, Chiba, Japan; 3 Department of Mental Health, AO San Carlo Borromeo, ASST Santi Paolo E Carlo, Milan, Italy; 4 Medical Research Council Brain Network Dynamics Unit, Nuffield Department of Clinical Neurosciences, University of Oxford, Oxford, UK; 5 Department of Neuropsychiatry, South West London and St George’s Mental Health Trust, London, UK; 6 Brighton and Sussex Medical School, University of Sussex, Brighton, UK; 7 Institute of Cognitive Neuroscience, UCL, London, UK; 8 Atkinson Morley Regional Neuroscience Centre, St George’s Hospital, London, UK; 9 Department of Clinical and Experimental Epilepsy, Institute of Neurology, UCL, National Hospital for Neurology and Neurosurgery, UCLH, Epilepsy Society, London, UK

**Keywords:** Neuroscience, Neurology

## Abstract

Interoception, the sense of the internal physiological state of the body, theoretically underpins aspects of self-representation. Experimental studies link feelings of body ownership to interoceptive perception, yet few studies have tested for association between the sense of agency and interoceptive processing. Here, we combined an intentional binding paradigm with cardiac measures of interoceptive processing (behavioural performance on a heartbeat discrimination task, and effects of timing within the cardiac cycle) in twenty-six non-clinical participants as an exploratory study. We found performance accuracy on the heartbeat discrimination task correlated positively with the intentional binding effect, an index of sense of agency (β = 0.832, p = 0.005), even after controlling for effects of age, sex, educational level, heart rate, heart rate variability and time accuracy. The intentional binding effect was enhanced during cardiac systole (compared to diastole) in individuals with greater heartbeat discrimination accuracy (β = 0.640, p = 0.047). These findings support the proposal that interoception contributes to mechanisms underlying the emergence of sense of agency.

## Introduction

The sense of a unitary, minimal (biological) self is a fundamental trait of human experience. Two core aspects of selfhood are proposed: The sense of ownership and the sense of agency^1^. The sense of ownership is the sense that “I” am the one who is undergoing an experience. The sense of agency is the sense that “I” am the one who is causing or generating an action and its effect^2^. Abnormalities in these fundamental aspects are reported in patients with neurological and psychiatric disorders, including sense of agency abnormalities in patients with functional movement disorder (FMD) and schizophrenia^2–6^. However, the mechanisms underpinning sense of agency in healthy individuals has yet to be completely clarified. Interoception refers to the central processing and representation of internal bodily signals, such as heartbeats and respiration^7^. Interoceptive signaling is a key component of homeostatic and allostatic control: The brain’s representation of changes in the internal physiological environment is necessary to evoke compensatory (e.g. autonomic reflexes, motor or behavioural) responses. In contrast, exteroceptive (e.g. visual and auditory) sensing refers to processing and representation of information in and about the external environment^7^. Interoception contributes to the evaluation of whether a change has an endogenous or exogenous origin^8^, and is therefore critical to distinguishing ‘self’ from ‘other’^9^. Indeed, convergent evidence supports the notion that interoception contributes to the sense of ownership^10–12^. Conversely, interoceptive differences are observed in patients with dissociative symptoms, who also experience aberrant sense of ownership^13–15^, although we found that patients with FMD have a normal SoO by means of the rubber hand paradigm^16^.

In contrast to sense of ownership, there is a paucity of direct evidence regarding the relationship between interoception and sense of agency^17^. However, individual differences in interoceptive sensitivity, indexed by performance accuracy on a heartbeat counting task, were found to correlate positively with self-reported sense of agency in a virtual-reality based paradigm^18^. Similarly, temporal judgement during a decision-making task was influenced by the phase of the cardiac cycle. This implicit measure of sense of agency was greater when the actions were implemented during the systolic period (during interoceptive baroreceptor signaling of heartbeat) compared to the diastolic period (between heartbeats)^19^. Furthermore, empirical studies show a relationship between interoception and action control^20,21^, which is related to the emergence of sense of agency^2^: Individual differences in heartbeat counting task performance accuracy predicted the ability to detect of errors in actions^21^. Furthermore, the systolic, compared to diastolic, period of the cardiac cycle was associated with better response inhibition on a stop-signal task, indicating that cardiac interoceptive signals exert a positive influence on action control^20^. Together these findings suggest that interoception contributes to the control of one’s own actions.

Finally, sense of agency and sense of ownership have been shown to be related to one another in the development of the sense of minimal self^18,22^. Studies using active rubber hand illusion (RHI) paradigms show strong correlations between self-report measures of perceived sense of agency and sense of ownership, more-so than between implicit, objective measures^23–26^. In one variation of the RHI, incorporating an intentional binding paradigm, a bi-directional relationship was observed between ownership of the rubber hand and indices of agency^22^. The illusory sense of ownership was promoted by the sense of agency, and sense of agency was modulated by the measured degree of embodiment of the rubber hand. Speculatively, this effect may arise as a result of the brain testing predictions about the internal or external source of sensory events through ‘active inference’ by moving the body, hence sharpening one’s own bodily boundaries^22^. Similarly, the relationship between sense of agency and sense of ownership may be mediated by interoception.

Together, these findings suggest that heightened cardiac interoception may positively enhance the development of sense of agency. In our study, interoceptive ability was assessed using two behavioural tests of heartbeat perception, the heartbeat tracking task (HTT) and the heartbeat detection task (HDT)^7^. We therefore hypothesised that interoceptive performance accuracy, obtained via these behavioural tests of heartbeat detection ability, would correlate with measures of sense of agency, as indexed implicitly using in the intentional binding task^2^. We also hypothesised that there would be a dissociation in the relationship between different measures of interoceptive ability (i.e. heartbeat tracking vs heartbeat discrimination task) and measures of sense of agency. We hypothesized that interoceptive accuracy assessed by the HDT, which assesses an individual’s ability to integrate interoceptive and exteroceptive information (in contrast to HTT), would specifically predict the intentional binding effect, since the consequences of actions in the intentional binding task are fed back via exteroceptive channels. Finally, we also hypothesized that cardiac interoceptive signaling may enhance or perturb sense of agency, depending on individual’s ability to integrate interoceptive and exteroceptive signals.

## Results

### Interoception and intentional binding effect

**Figure 1:**
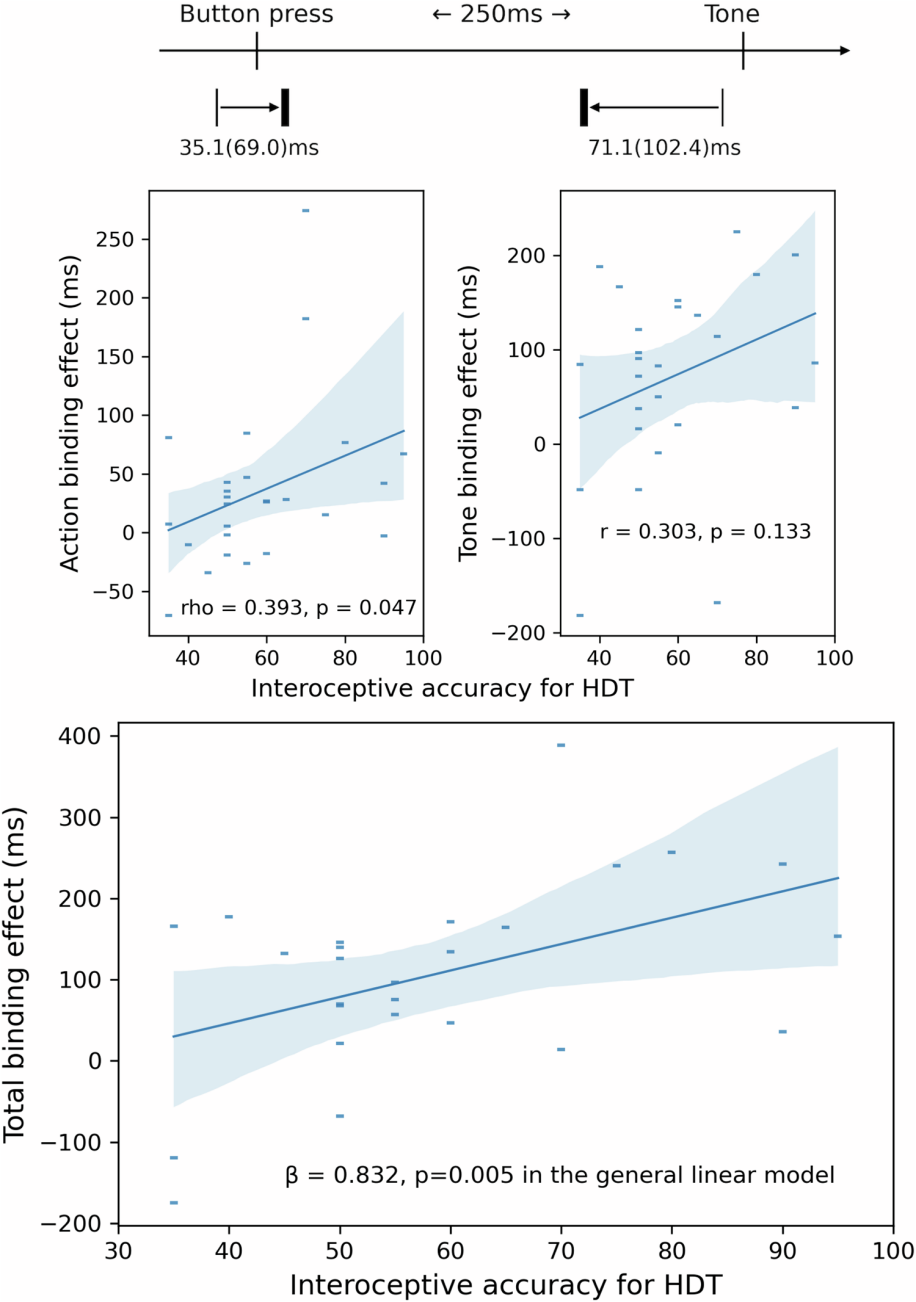
Total intentional binding effect was significantly predicted by interoceptive accuracy for HDT (β = 0.832, p = 0.005).

Interoceptive performance accuracy for the HTT was mean (SD) = 0.62 (0.25). Interoceptive accuracy for the HDT was mean (SD) = 0.58 (0.17). Metacognitive interoceptive ‘awareness’ calculated from trial-by-trial correspondence between accuracy and confidence in performing the HDT was mean (SD) = 0.56 (0.14). Interoceptive accuracy for HTT and HDT were significantly correlated (r = 0.455, p = 0.019). Interoceptive awareness calculated from HDT was also significantly correlated with interoceptive accuracy for HDT (r = 0.535, p = 0.005), and had a trend level correlation with interoceptive accuracy for HTT (r = 0.350, p = 0.079). The participants also performed a time tracking task (TTT) as a control task for the HTT (Koreki et al. 2020). TTT performance accuracy score was mean (SD) = 0.81 (0.15) and was not associated with interoceptive accuracy for either task, or interoceptive awareness (all p > 0.05). On the intentional binding task, the action binding effect score across participants was mean (SD) = 35.1 (69.0) ms and the tone binding effect score was mean (SD) = 71.1 (102.4) ms. Total binding effect score was mean (SD) 106.2 (118.8) ms (Fig. 1). Time accuracy was not associated with any binding effects (all p > 0.05).

### Binding effect and the relationship to interoception

Accuracy for HDT was significantly correlated with total binding effects (r = 0.461, p = 0.018). Accuracy for HTT and awareness for HDT were not correlated with total binding effects (p = 0.383, 0.176, respectively). With regards to each binding effect, accuracy for HDT was significantly correlated with action binding effects (rho = 0.393, p = 0.047), but not with tone binding effects (r = 0.303, p = 0.133) (Fig. 1—top panel). Accuracy for HTT had a trend level correlation with action binding effects (rho = 0.352, p = 0.078), but not with tone binding effects (p = 0.874). Awareness for HDT was significantly correlated with action binding effects (rho = 0.482, p = 0.013), but not with tone binding effects (p = 0.630). Our statistical model (Akaike’s information criterion (AIC) = 72.5), revealed that the total binding effect was significantly predicted by interoceptive accuracy for HDT (β = 0.832, standard error (SE) = 0.256, t = 3.248, p = 0.005), as well as age (β = 0.532, SE = 0.211, t = 2.523, p = 0.023) and time accuracy (β = 0.482, SE = 0.482, t = 2.242, p = 0.040) (Table 1, Fig. 1—bottom panel).

**Table 1:** General linear models for binding effects.

	β*	SE	t-values	p-values
**Total binding effect**				
(Intercept)	− 0.087	0.263	− 0.330	0.746
**Age**	0.532	0.211	2.523	**0.023**
Female	0.150	0.362	0.415	0.683
Education	− 0.178	0.194	− 0.922	0.370
HTT accuracy	− 0.410	0.225	− 1.827	0.086
**HDT accuracy**	0.832	0.256	3.248	**0.005**
HDT awareness	0.199	0.214	0.928	0.367
**TTT accuracy**	0.482	0.215	2.242	**0.039**
Heart rate	0.245	0.260	0.942	0.360
RMSSD	0.224	0.220	1.019	0.323

**The difference of total binding effect between systole and diastole**				
(Intercept)	− 0.058	0.305	− 0.191	0.851
Age	0.175	0.245	0.713	0.486
Female	0.101	0.420	0.240	0.813
Education	0.161	0.225	0.715	0.485
HTT accuracy	− 0.322	0.261	− 1.233	0.235
**HDT accuracy**	0.640	0.297	2.151	**0.047**
HDT awareness	0.072	0.249	0.291	0.775
TTT accuracy	− 0.045	0.250	− 0.181	0.859
Heart rate	0.346	0.302	1.145	0.269
RMSSD	0.361	0.255	1.413	0.177

*SE* Standard error, *HTT* Heartbeat Tracking Task, *HDT* Heartbeat Discrimination Task, *TTT* Time Tracking Task, *RMSSD* Root Mean Square of the Successive Differences. *Standardised values.

### Overview regarding differences between systole and diastole

There was no significant difference of total binding effects between systole and diastole (99.1 (131.4) in systole vs 108.3 (115.0), p = 0.328) (Table 2), as well as any other relevant values [Action only, − 16.1 (77.0) in systole vs − 22.2 (74.3), p = 0.182; Tone only, − 14.6 (69.4) in systole vs − 9.7 (66.8), p = 0.409; Action of Action-Tone condition, 17.3 (63.1) in systole vs 13.8 (62.9), p = 0.256; Tone of Action-Tone condition, − 80.2 (123.8) in systole vs − 82.0 (131.8), p = 0.764; Action binding, 33.5 (72.4) in systole vs 36.0 (69.5), p = 0.694; Tone binding, − 65.6 (106.7) in systole vs − 72.3 (101.5), p = 0.400].

**Table 2:** Effects of heartbeats on agency regardless of interoception.

Condition	Action time	paired-T	Condition	Tone time	paired-T
**Action only**			**Tone only**		
Systole	− 16.1 (77.0)	t = 1.372, p = 0.182	Systole	− 14.6 (69.4)	t = − 0.840, p = 0.409
Diastole	− 22.2 (74.3)		Diastole	− 9.7 (66.8)	
**Action-Tone**			**Action-Tone**		
Systole	17.3 (63.1)	t = 1.162, p = 0.256	Systole	− 80.2 (123.8)	t = 0.304, p = 0.764
Diastole	13.8 (62.9)		Diastole	− 82.0 (131.8)	
**Action binding**			**Tone binding**		
Systole	33.5 (72.4)	t = − 0.399, p = 0.694	Systole	− 65.6 (106.7)	t = − 0.857, p = 0.400
Diastole	36.0 (69.5)		Diastole	− 72.3 (101.5)	
***Total binding***					
Systole	99.1 (131.4)	t = − 0.999, p = 0.328			
Diastole	108.3 (115.0)				

### Influence of cardiac phase on binding effects

**Figure 2:**
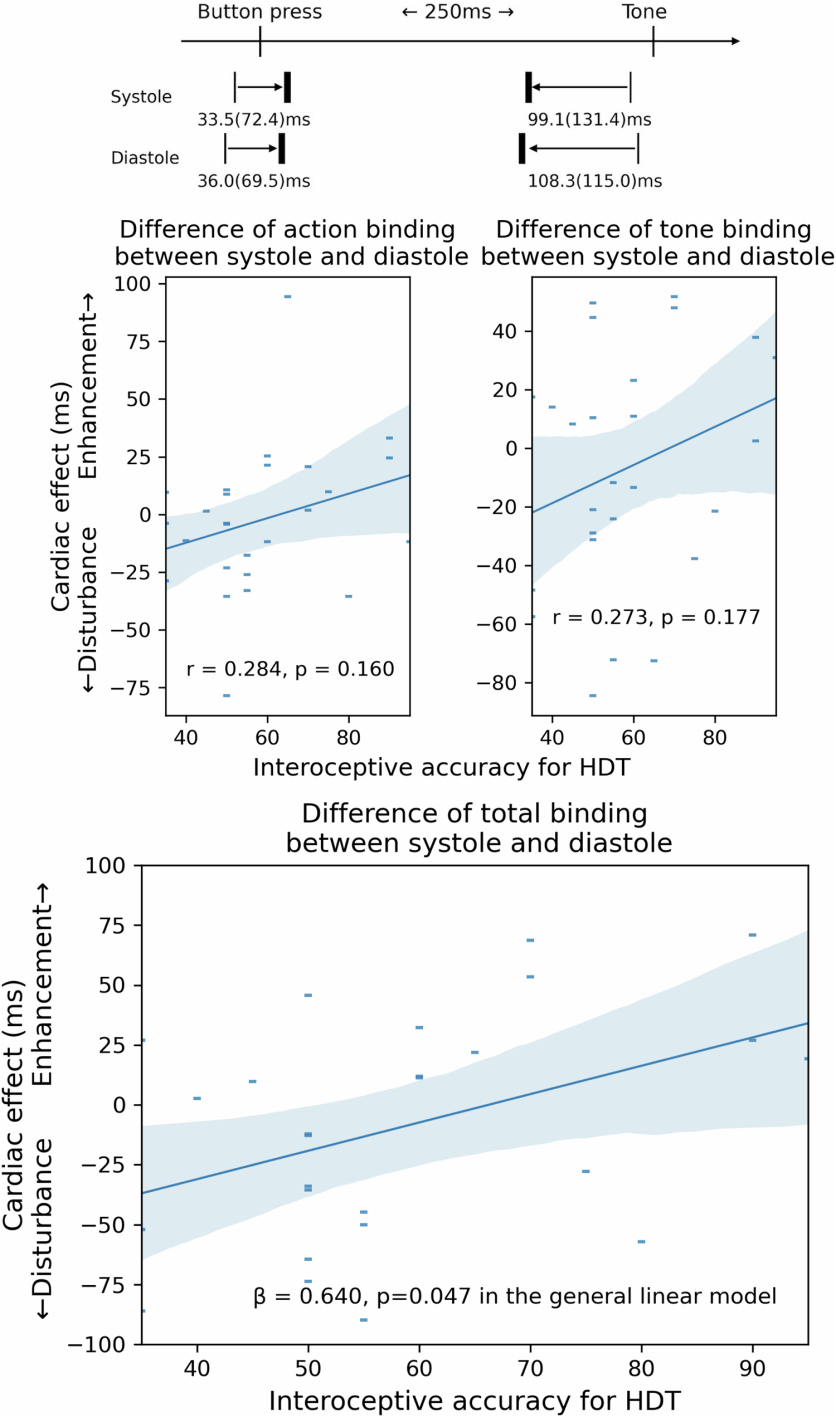
Cardiac effect can enhance or disturb total binding effects depending on interoceptive accuracy for HDT (β = 0.640, p = 0.047).

The correlation analysis revealed that an effect of the phase of the cardiac cycle (i.e. the difference between systole and diastole) on total binding was significantly correlated with accuracy for HDT (r = 0.424, p = 0.031), but not accuracy for HTT (p = 0.603), nor awareness for HDT (p = 0.438). Accuracy for HDT was not significantly correlated with an effect of the phase of the cardiac cycle on either action binding (r = 0.284, p = 0.160), nor tone binding (r = 0.273, p = 0.177) (Fig. 2—top panel). Accuracy for HTT was not significantly correlated with an effect of the phase of the cardiac cycle on either action binding (p = 0.466), nor tone binding (p = 0.231). Awareness for HDT was not significantly correlated with an effect of the phase of the cardiac cycle on either action binding (p = 0.892), nor tone binding (p = 0.306). Our statistical model (AIC = 80.3), revealed that an effect of the phase of the cardiac cycle on total binding was only significantly predicted by interoceptive accuracy for HDT (β = 0.640, SE = 0.297, t = 2.151, p = 0.047) (Table 1, Fig. 2 bottom panel).

### Sensitivity analysis

In the sample including outliers, accuracy for HDT was significantly correlated with the total binding effect (r = 0.467, p = 0.012), and was also significantly correlated with the effect of the phase of the cardiac cycle on the total binding effect (rho = 0.462, p = 0.013). Neither accuracy for HTT nor awareness for HDT was correlated with total binding effects (p = 0.377, 0.206, respectively) nor with the effect of the phase of the cardiac cycle on the total binding effect (p = 0.766, 0.646, respectively).

In a forced entry general linear model, there was a significant effect of interoceptive accuracy for HDT on the total binding effects (β = 0.888, SE = 0.268, t = 3.314, p = 0.004) with trend level association of age (β = 0.433, SE = 0.208, t = 2.088, p = 0.051) and time accuracy (β = 0.423, SE = 0.223, t = 1.896, p = 0.074), and there was a trend-level effect of interoceptive accuracy for HDT on the effect of the phase of the cardiac cycle (β = 0.577, SE = 0.294, t = 1.960, p = 0.066). To address the issue of excessive and similar independent variables, stepwise regression methods were also conducted. Interoceptive accuracy for HDT and age were selected in the final model for the total binding effect, revealing a significant effect of interoceptive accuracy for HDT (β = 0.485, SE = 0.161, t = 3.012, p = 0.006) as well as age (β = 0.368, SE = 0.161, t = 2.285, p = 0.031). Interoceptive accuracy for HDT, HR and RMSSD were selected in the final model for the effect of the phase of the cardiac cycle on total binding, revealing a significant effect of interoceptive accuracy for HDT on the effect of the phase of the cardiac cycle (β = 0.449, SE = 0.190, t = 2.357, p = 0.027), but no effect of HR (p = 0.747). and RMSSD (p = 0.915).

## Discussion

This is the first report to investigate the relationship between cardiac interoception and sense of agency. In accordance with our hypothesis, the intentional binding effect, an implicit index of sense of agency, was significantly predicted by interoceptive performance accuracy on the HDT. We also found that the binding effect was influenced by the phase of the cardiac cycle (systole > diastole) as a function of increasing HDT performance accuracy. Our sensitivity analyses showed similar results, although outlying participants may affect some results and more robust evidence should be provided from future larger studies. These findings cannot be explained by increased accuracy of perceptual judgement in both tasks because a stronger binding effect paradoxically means reduced accuracy or reduced correspondence between subjective and objective button press/tone estimates. In the intentional binding task, the temporal gap between subjective and objective times at the timing of action or tone is calculated. Here, a smaller gap even in the action-tone condition reflects increased accuracy, but also a weaker binding effect. On the other hand, a bigger gap in the action-tone conditions reflects reduced accuracy, but also a stronger binding effect.

The effects of interoception on the intentional binding may have several potential mechanisms. Firstly, the previously reported enhancement of sense of ownership by interoception may also exert a positive effect on sense of agency. Correspondingly, it has been shown that cardiac feedback synchronized with visual feedback enhances the sense of ownership in the rubber hand paradigm^11^. Similarly, cardiac feedback also improves sense of ownership using full body illusion paradigms^10^. Together, these findings emphasize the importance of cardiac-related perceptions to the development of sense of ownership. In addition, there is accumulating evidence for positive interplay between sense of ownership and sense of agency^18,22^. In the rubber hand illusion, self-reported sense of agency based on questionnaire ratings was higher when the model hand was anatomically congruent (higher sense of ownership) compared to when it was anatomically incongruent^25^. Thus the sense of ownership may mediate the link between cardiac interoception and the sense of agency. A second potential mechanism, is that interoception may enhance the precision of appropriate action control in forward models of action, leading to enhancement of sense of agency. Action selection is proposed to have a positive effect of on sense of agency^2^. Here, the ability to select which action to make can increase the likelihood that actual consequences of actions will match the desired consequences. Sense of agency is the attribution of oneself as the cause of one’s own actions and consequences of actions. Hence in the forward model of motor control, a close match between predicted and actual consequences of action will enhance sense of agency. More accurate cardiac interoception may decrease noise (increasing precision) in action selection and control, thereby enhancing sense of agency.

In the intentional binding task, the consequence of action was delivered to the brain as an exteroceptive input. Total binding was influenced by interoception, suggesting a positive effect on the processing of exteroceptive feedback regarding the consequence of action. The HDT assesses not just interoceptive accuracy, but the ability to integrate interoception and exteroception, unlike the HTT. We observed that interoceptive performance accuracy on the ‘crossmodal’ HDT (but not HTT) was associated with this effect. This observation implies that both the accurate ascertainment of internal physiological signals and the capacity to integrate interoceptive with exteroceptive information (particularly the perception of action consequences), is important for the emergence of sense of agency. In a predictive coding framework related to this account, interoceptive ‘predictions’ are suggested to contribute to sense of agency via their integration with predicted and actual sensory feedback from action^17^. One paper conducted as Ph.D. theme of Royal Holloway, University of London, showed a significant positive relationship between interoceptive accuracy measured by HTT and the binding effect^27^. Unlike their finding, while we found a significant positive correlation between HTT and HDT, there was no significant association between HTT accuracy and the binding effect in our study. One possible factor causing this difference in findings is the age of participants. Their participants were students, and their mean age was 20.3. Age may affect the binding effects^28^. Indeed, our data showed a significant effect of age on the binding effect. Although our participants were more heterogeneous than that of their study, age was controlled in our analysis, and our results may therefore be more generalisable. In addition, notwithstanding the risk of lower sensitivity due to age heterogeneity in detecting an association between HTT accuracy and binding in our study, we found that HDT accuracy was more strongly associated with the binding effect than HTT accuracy.

We also observed a cardiac timing effect on sense of agency that was dependent upon individual profiles in interoceptive perceptual sensitivity to heartbeats, whereby individuals with greater HDT performance accuracy showed enhanced binding at cardiac systole compared to diastole. At systole compared to diastole, arterial baroreceptors signal to the brain the strength and timing of individual heartbeats. Here we show that such cardiac interoceptive signals influence sense of agency in people with greater perceptual attunement to their own heartbeats. This finding is in line with one published study^19^, but our current study adds to this evidence by showing that interoceptive signals at systole can disturb sense of agency in individuals with imprecise interoceptive perceptual abilities (low HDT performance accuracy) yet enhance sense of agency in individuals with precise interoceptive perceptual abilities. Cardiac timing effects (systolic vs diastolic phase of the cardiac cycle) have been shown to modulate a number of cognitive, emotional and perceptual processes: In studies of action control, motor response inhibition in a stop-signal task is observed to greater during systole compared to diastole, suggesting that heartbeat-triggered interoceptive signals aid action inhibition^20^. Other groups also report faster responses and better response inhibition in similar non-emotional tasks during the systolic period compared to the diastolic period^29^. Herman and Tsakiris study demonstrated the intentional binding effect was greater when actions were done during systole than diastole^19^. Unlike their findings, no general difference regardless of interoceptive accuracy was found in the present study, possibly due to differences in participant characteristics and experimental settings. Further studies are required to further explore this aspect. They also found that binding was weaker when both actions and its effect were carried out during systole, or both during diastole. In the present study, the interval between action and tone was 250 ms, which was too short to have both action and tone set during systole, and we could therefore not analyse this point. Based on our findings, we suggest that the heartbeat has a significant bi-directional influence on sense of agency, contingent on interoceptive accuracy.

Our findings have clinical relevance. A range of neuropsychiatric disorders, including FMD and schizophrenia, are associated with both abnormalities of interoception and sense of agency, potentially linking directly to important clinical phenomena. Patients with FMD manifest pathologically low sense of agency. Previous work by our group has also independently demonstrated poor interoception and poor sense of agency in patients with FMD^5,6,14,30^. Thus, our findings in the present study highlight the positive relationship between interoception and sense of agency, to suggest that poor interoception may underpin the reduced sense of agency observed in patients with FMD. Furthermore, acute and chronic stress are common in patient with FMD^31^ and a stress-associated increase in heart rate may thus further compromise sense of agency in these patients as a function of the (systolic) cardiac timing effects on sense of agency that we identified in this study. In contrast, there may be a complex relationship between interoception and sense of agency in schizophrenia, who show poor interoception but experimentally excessive sense of agency^2–4,32–34^. Haggard, et al. explained excessive sense of agency from the standpoint of retrospective inference^2^. In addition, it was demonstrated that the relationship between interocepton and emotional face recognition in schizophrenia was opposite to any other conditions including in healthy people^35^. Further study is required to understand this complex relationship in schizophrenia.

There are several limitations to this study. First, our sample size is relatively small, possibly leading to insufficient power to detect other significant associations. In addition, the modeling of a large number of independent variables and similar parameters has statistical limitations. Although a stepwise model has a risk of Type I error, both regression models showed similar results. Our findings are preliminary and a further larger study is required to replicate and extend these findings. Second, the present study is cross-sectional and correlational hence causal inferences cannot be drawn. Given that interoception plays a more fundamental role than simply discriminating between self and others, it may therefore contribute to mechanisms underlying the emergence of sense of agency, as well as the emergence of perception from inside the body. However, we cannot completely rule out the opposite effect, namely that emergence of the sense of agency may contribute to interoception*.* An interventional study is necessary to investigate causality. Third, in the present study, all participants first completed the interoceptive task and then the intentional binding task. Therefore, it is possible that our results are confounded by an order effect. Due to practical problems (including the attachment of ECG in the intentional binding task), we could not manage the order to mitigate this issue. While, as noted earlier, our findings cannot be explained by increased accuracy of perceptual judgement in both tasks, a more randomized experiment is required. Fourth, the ‘classic’ intentional binding task is insufficient to assess effects of interoception on the prospective and retrospective components of sense of agency^2^. A further study using a modification of the task, with varying probability of tone occurrence is necessary to assess these components. Fifth, because only non-clinical participants were recruited, a study in patients is necessary to test our hypotheses about the pathophysiology of FMD. Sixth, although our study was based on the classical interpretation of intentional binding effects (i.e. sense of agency), there is still debate about the meaning of this effect^2,36^.

In conclusion, the intentional binding effect, an implicit measure of sense of agency, was stronger in individuals with increased cardiac interoceptive accuracy (on the HDT), who moreover show an enhancement of intentional binding effects at cardiac systole, in contrast to individuals with lower cardiac interoceptive ability. These findings shed light on mechanisms potentially underlying the pathophysiology of specific neurological and psychiatric illness conditions, including FMD in which patients manifest both poor interoception and reduced sense of agency.

## Methods

### Participants

Thirty-three people were recruited as self-reported healthy individuals. As our study was exploratory, sample size estimation was not strictly conducted and sample size in the present study was determined based on our previous associated papers^14,20^. Three people later reported chronic illnesses (hypertension, migraine, and well-controlled epilepsy) on questioning and that they were taking medications. These individuals were also excluded from the main analysis. Two additional people were excluded due to technical failure of electrocardiogram (ECG) recordings at the time of experimental testing. Of the remaining individuals, two participants were outliers in respect of performance on HTT accuracy and the difference of total binding between systole and diastole (as determined with a threshold of mean ± 3sd applied to all participants across the main tasks including HDT/HTT accuracy, total binding effect and cardiac difference in total binding), and were excluded. Finally, twenty-six individuals (mean (SD) age: 33.3 (10.2) years old, female: n = 15, educational level (SD): 18.7 (2.9) years, right handedness: n = 24, heartrate: 72.5 (11.6)) were therefore included in the main analysis. The data from these 26 participants is plotted in both top and bottom panels of each Figure. In addition, the data of 28 participants (including the two participants classified as outliers) were analysed as part of a sensitivity analysis of our results. Written informed consent was obtained. This study was approved by the St George’s Research Ethics Committee. All methods were performed in accordance with the relevant guidelines and regulations.

### Tasks

All tasks were operated by a laptop computer. Participants were firstly asked to complete the interoception tasks (HTT followed by the HDT), and then asked to complete the intentional binding task.

### Interoception tasks

During the two interoceptive tasks, participants’ heartbeats were monitored via a medical-grade pulse oximeter (Nonin Xpod 3012LP) with the sensor mounting attached to their index finger of their non-dominant hand. Their interoceptive accuracy and awareness were assessed by a heartbeat tracking task (HTT) and a heartbeat discrimination task (HDT)^7^.

In the HTT, participants were required to count their own heartbeats, without touching any body part, during six time windows (randomized trials of 25, 30, 35, 40, 45 and 50 s.), and they were asked to report the number of heartbeats detected to an experimenter at the end of each trial. Interoceptive accuracy for HTT was calculated on a trial-by-trial basis based on the ratio of perceived to actual heartbeats: 1 – (nbeats_real_ – nbeats_reported_)/((nbeats_real_ + nbeats_reported_)/2), and these were averaged to form a mean score^7^. This formula calculates interoceptive accuracy independent of the amount of heartbeats in the trial by normalizing the absolute error in perceived heartbeats as a function of the overall number of heartbeats.

In the HDT, participants were asked to listen on each trial to ten tones and then indicate their judgement whether the tones were in synchrony with, or delayed relative to, their own heartbeat. There were 20 trials in total. Each trial consisted of ten tones presented at 440 Hz and having 100 ms duration, which were triggered by the heartbeat. Under the synchronous condition, tones were triggered by onset of the finger pulse waveform (i.e. on average ~ 250 ms after the R-wave hence concurrent with ventricular systolic emptying). Under the delayed condition, an additional delay of 300 ms was inserted and tones were thus presented at ~ 550 ms after the R-wave. These tones under synchronous or asynchronous conditions correspond to maximum and minimum synchronicity judgements respectively (e.g.^37,38^). At the end of each trial, they were asked about whether they experienced the tones to be synchronous or asynchronous with their own heartbeats. Interoceptive accuracy for HDT was calculated as a ratio of correct to incorrect synchronicity judgments. They were also asked to score their confidence in the synchronicity judgment on a visual analogue scale ranging from total guess to complete confidence in each trial. Metacognitive interoceptive ‘awarenes’s (insight) was calculated using a receiver operating characteristic (ROC) analysis to determine the diagnostic significance of confidence for accuracy on a trial-by-trial basis. Area under the ROC curve denoted the degree to which confidence is predictive of accuracy and it was defined as a value of interoceptive awareness for HDT.

As a control task for the HTT, they are asked to complete a time tracking task (TTT) where they were required to judge the number of seconds of the six randomized various time windows, following the same procedure as in the HTT. Time accuracy was also calculated in the similar way to performance accuracy on the HTT.

### Intentional binding task

In a computerized task, participants were asked to look at a clock on a computer screen and press a space key with their index finger of dominant hand in accordance with each condition^2^. There were 4 conditions and 40 trials in each condition. The order of the conditions was randomized. In the first condition (action-only condition), participants were required to press a button once during the second rotation of the clock hand and report the clock hand position at which they pressed the button. In the second condition (tone-only condition), they were required to listen for a tone and report the clock hand position at which they heard the tone. In the third and fourth condition (action-tone condition), they were required to press the button in the same way as the first condition, but their button press caused a tone 250 ms after their press. In the third condition, they were required to report the clock hand position at which they pressed the button as per the first condition. In the fourth condition, they were required to report the clock hand position at which they heard the tone as per the second condition. The action binding effect was defined as a subtraction of reported time between the first and third conditions. The tone binding effect was defined as a subtraction of reported time between the second and fourth conditions. The total binding effect was calculated as the addition of both binding effects. During this intentional binding task, participants were attached to an electrocardiograph (ECG), which was a part of BIOSEMI EEG system. Two electrodes were attached on both upper arms and the placement of these electrodes were adjusted to avoid muscle artefacts during the task and to show clear R waves.

### Data analysis

Statistical analyses were carried out using R (4.0.2). A general linear model was performed using a multiple linear regression model with total binding effect as the dependent variable. The following independent variables were included in our model; age, sex, education, interoceptive accuracy for HTT, interoceptive accuracy for HDT, interoceptive awareness, accuracy for TTT, HR during tasks, and heart rate variability (Root Mean Square of the Successive Differences: RMSSD) during tasks. Furthermore, to explore whether action and/or tone binding was associated with interoceptive accuracy for HDT, simple parametric or non-parametric correlational analyses were performed depending on the normality of the data based on the Shapiro–Wilk test.

The definition of whether the button press and tone in each trial occurred during the systolic or diastolic period was based on post hoc assessment of the ECG recording after the completion of the tasks. As in previous studies, the systolic period was defined as the period of 290 ± 100 ms after R-wave^20^. Action-only trials were divided into trials with actions during systolic or diastolic periods. Similarly, action-tone trials with reports about button press (the third condition) were divided into trials with actions during systolic or diastolic period. The systolic action binding effect was defined as the subtraction of the reported time of trials with action during the systolic period between the first and third conditions. The diastolic action binding effect was defined as the subtraction of the reported time of trials with actions during the diastolic period between the first and third conditions. Finally, the cardiac effect on action binding was defined as a subtraction between systolic and diastolic action binding effect.

In a similar manner, tone-only trials and action-tone trials with reports about tones (the fourth condition) were divided into trials with tones during systolic or diastolic period. The systolic tone binding effect was defined as a subtraction of reported time of trials with tones during systolic period between the second and fourth conditions. The diastolic tone binding effect was defined as the subtraction of reported time of trials with tones during diastolic period between the second and fourth conditions. Finally, the cardiac effect on tone binding was defined as the subtraction between the systolic and diastolic tone binding effect.

The total cardiac effect was defined as an addition of both cardiac effects. A general linear model was performed with total cardiac effect as the dependent variable and same independent variables as above. In the model, variance inflation factors were small enough to deny a risk of multicollinearity. Residuals of these models were normally distributed based on the Shapiro–Wilk test. Similarly, to explore the effect of HDT accuracy on cardiac modulation of action and/tone binding, simple parametric or non-parametric correlational analyses were performed depending on the normality of the data based on the Shapiro–Wilk test.

As part of a sensitivity analysis, we conducted two further analyses in addition to our main analysis. These analyses included two participants who were outliers. The first model was a forced entry model, which was same as our aforementioned main analysis. The second model included a forward–backward stepwise selection method to deal with the issue of an excessive number of, and similar, independent variables.

## Data Availability

The datasets generated during the current study are available from the corresponding author upon reasonable request, subject to regulatory approval.

## References

[1] Gallagher S (2000). Philosophical conceptions of the self: Implications for cognitive science. Trends Cogn. Sci..

[2] Haggard P (2017). Sense of agency in the human brain. Nat. Rev. Neurosci..

[3] Koreki A (2015). Behavioral evidence of delayed prediction signals during agency attribution in patients with schizophrenia. Psychiatry Res..

[4] Koreki A (2019). Dysconnectivity of the agency network in schizophrenia: A functional magnetic resonance imaging study. Front. Psychiatry..

[5] Kranick SM (2013). Action-effect binding is decreased in motor conversion disorder: Implications for sense of agency. Mov. Disord..

[6] Pareés I (2014). Loss of sensory attenuation in patients with functional (psychogenic) movement disorders. Brain.

[7] Critchley HD, Garfinkel SN (2017). Interoception and emotion. Curr. Opin. Psychol..

[8] Ceunen E, Vlaeyen JW, Van Diest I (2016). On the origin of interoception. Front. Psychol..

[9] Palmer CE, Tsakiris M (2018). Going at the heart of social cognition: Is there a role for interoception in self-other distinction?. Curr. Opin. Psychol..

[10] Heydrich L (2018). Cardio-visual full body illusion alters bodily self-consciousness and tactile processing in somatosensory cortex. Sci. Rep..

[11] Suzuki K, Garfinkel SN, Critchley HD, Seth AK (2013). Multisensory integration across exteroceptive and interoceptive domains modulates self-experience in the rubber-hand illusion. Neuropsychologia.

[12] Tsakiris M, Tajadura-Jiménez A, Costantini M (2011). Just a heartbeat away from one's body: Interoceptive sensitivity predicts malleability of body-representations. Proc. Biol. Sci..

[13] Garfinkel S (2018). Metacognitive deficits in interoception are associated with dissociative experiences in patients with first episode psychosis. Schizophrenia Bull..

[14] Koreki A (2020). Trait and state interoceptive abnormalities are associated with dissociation and seizure frequency in patients with functional seizures. Epilepsia.

[15] Sedeño L (2014). How do you feel when you can't feel your body? Interoception, functional connectivity and emotional processing in depersonalization-derealization disorder. PLoS ONE.

[16] Demartini B, Ricciardi L, Crucianelli L, Fotopoulou A, Edwards MJ (2016). Sense of body ownership in patients affected by functional motor symptoms (conversion disorder). Conscious Cogn..

[17] Marshall AC, Gentsch A, Schütz-Bosbach S (2018). The interaction between interoceptive and action states within a framework of predictive coding. Front. Psychol..

[18] Stern Y, Koren D, Moebus R, Panishev G, Salomon R (2020). Assessing the relationship between sense of agency, the bodily-self and stress: Four virtual-reality experiments in healthy individuals. J. Clin. Med..

[19] Herman AM, Tsakiris M (2020). Feeling in control: The role of cardiac timing in the sense of agency. Affec. Sci.

[20] Rae CL (2018). Response inhibition on the stop signal task improves during cardiac contraction. Sci. Rep..

[21] Sueyoshi T, Sugimoto F, Katayama J, Fukushima H (2014). Neural correlates of error processing reflect individual differences in interoceptive sensitivity. Int. J. Psychophysiol..

[22] Braun N (2018). The senses of agency and ownership: A review. Front. Psychol..

[23] Braun N, Thorne JD, Hildebrandt H, Debener S (2014). Interplay of agency and ownership: The intentional binding and rubber hand illusion paradigm combined. PLoS ONE.

[24] Dummer T, Picot-Annand A, Neal T, Moore C (2009). Movement and the rubber hand illusion. Perception.

[25] Kalckert A, Ehrsson HH (2012). Moving a rubber hand that feels like your own: A dissociation of ownership and agency. Front. Hum. Neurosci..

[26] Walsh LD, Moseley GL, Taylor JL, Gandevia SC (2011). Proprioceptive signals contribute to the sense of body ownership. J. Physiol..

[27] Ainley V. The Heartfelt Self: Investigating Interactions between Individual Differences in Interoceptive Accuracy and Aspects of Self-Processing. 2015. 207. https://pure.royalholloway.ac.uk/portal/en/publications/the-heartfelt-self(0fb7587e-3910-4e0d-ab55-e25952a0b543).html

[28] Aytemur A, Levita L (2021). A reduction in the implicit sense of agency during adolescence compared to childhood and adulthood. Conscious Cogn..

[29] Makowski D, Sperduti M, Blondé P, Nicolas S, Piolino P (2019). The heart of cognitive control: Cardiac phase modulates processing speed and inhibition. Psychophysiology.

[30] Ricciardi L (2016). Interoceptive awareness in patients with functional neurological symptoms. Biol. Psychol..

[31] Keynejad RC (2019). Stress and functional neurological disorders: Mechanistic insights. J. Neurol. Neurosurg. Psychiatry..

[32] Koreki A, Funayama M, Terasawa Y, Onaya M, Mimura M (2021). Aberrant interoceptive accuracy in patients with schizophrenia performing a heartbeat counting task. Schizophrenia Bull. Open.

[33] Ardizzi M (2016). Interoception and positive symptoms in schizophrenia. Front. Hum. Neurosci..

[34] Torregrossa LJ, Amedy A, Roig J, Prada A, Park S (2022). Interoceptive functioning in schizophrenia and schizotypy. Schizophr. Res..

[35] Critchley HD (2019). Transdiagnostic expression of interoceptive abnormalities in psychiatric conditions. MedRxiv.

[36] De Pirro S (2020). Effect of alcohol on the sense of agency in healthy humans. Addict Biol..

[37] Wiens S, Palmer SN (2001). Quadratic trend analysis and heartbeat detection. Biol. Psychol..

[38] Sophie B (2021). Evidence toward the potential absence of relationship between temporal and spatial heartbeats perception. Sci. Rep..

